# Sequential Observations of Conversion from Nonischemic to Ischemic Central Retinal Vein Occlusion Using Optical Coherence Tomography Angiography

**DOI:** 10.1155/2018/1354217

**Published:** 2018-04-23

**Authors:** Akira Fukutomi, Kotaro Tsuboi, Hikari Ono, Yuichiro Ishida, Motohiro Kamei

**Affiliations:** Department of Ophthalmology, Aichi Medical University, Nagakute, Japan

## Abstract

We report the sequential changes of retinal vessels observed by optical coherence tomography angiography (OCTA) in a case of nonischemic central retinal vein occlusion (CRVO) that converted to ischemic CRVO. An 81-year-old woman visited our Retina Clinic because of visual acuity loss in the left eye. Funduscopic examination showed venous tortuosity and intraretinal hemorrhage in all four quadrants of the fundus. OCT showed macular edema. Fluorescein angiography (FA) and OCTA showed loss of small capillaries. Nonischemic CRVO was diagnosed. Antivascular endothelial growth factor (VEGF) treatment resolved the edema and improved visual acuity. However, during follow-up, capillary dropout was observed on OCTA, which gradually enlarged. Eventually, FA confirmed the conversion to ischemic CRVO. In this case, sequential observations using OCTA showed that nonischemic CRVO did not convert to ischemic CRVO abruptly but occurred stepwise. Additionally, vascular changes began around the veins and blood flow changes were observed more clearly in deep capillary plexus than in superficial capillary plexus.

## 1. Introduction

Central retinal vein occlusion (CRVO) is a significant cause of acquired vision loss [1]. CRVO was traditionally classified into ischemic and nonischemic subtypes on the basis of the degree of retinal capillary nonperfusion. These two different types have very different outcomes. Ischemic CRVO, defined angiographically as showing at least 10-disc area of retinal capillary nonperfusion, has worse vision at initial presentation and at follow-up than nonischemic CRVO [2]. In ischemic CRVO, neovascular glaucoma (NVG) develops in at least 23% of the eyes after 15 months [3]. However, in nonischemic CRVO, development of NVG is rare. It is well known that eyes with nonischemic CRVO may convert to ischemic CRVO during follow-up. The Central Vein Occlusion Study (CVOS) reported a conversion rate from nonischemic CRVO to ischemic CRVO of 3.3% by 4 months after study entry and an incidence rate 10 times higher by 3 years [2]. In nonischemic CRVO, due to potential serious complications, frequent follow-up is needed. Recently, optical coherence tomography angiography (OCTA) has allowed visualization of microvascular abnormalities without dye injection and considered it useful for frequent follow-ups. Furthermore, OCTA has been reported that it is useful to identify eyes with low vascular densities which are at high risk of neovascular complications [4]. We report sequential changes of retinal perivascular capillaries observed using OCTA in a case of nonischemic CRVO that converted to ischemic CRVO. OCTA images were obtained using the RTVue XR Avanti (Optovue, Inc., Fremont, CA, USA) and Cirrus HD-5000 AngioPlex (Ziess, Inc., Oberkochen, Germany).

## 2. Case Report

**Figure 1:**
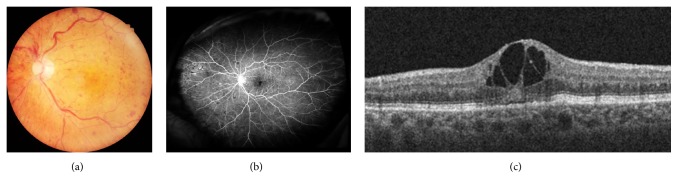
Image findings in the left eye at initial visit. (a) Fundus photograph demonstrating venous tortuosity and intraretinal hemorrhage in all four quadrants of the funds. (b) Fluorescein angiography demonstrating an intact parafoveal capillary network. (c) Optical coherence tomography demonstrating inner retinal cysts.

**Figure 2:**
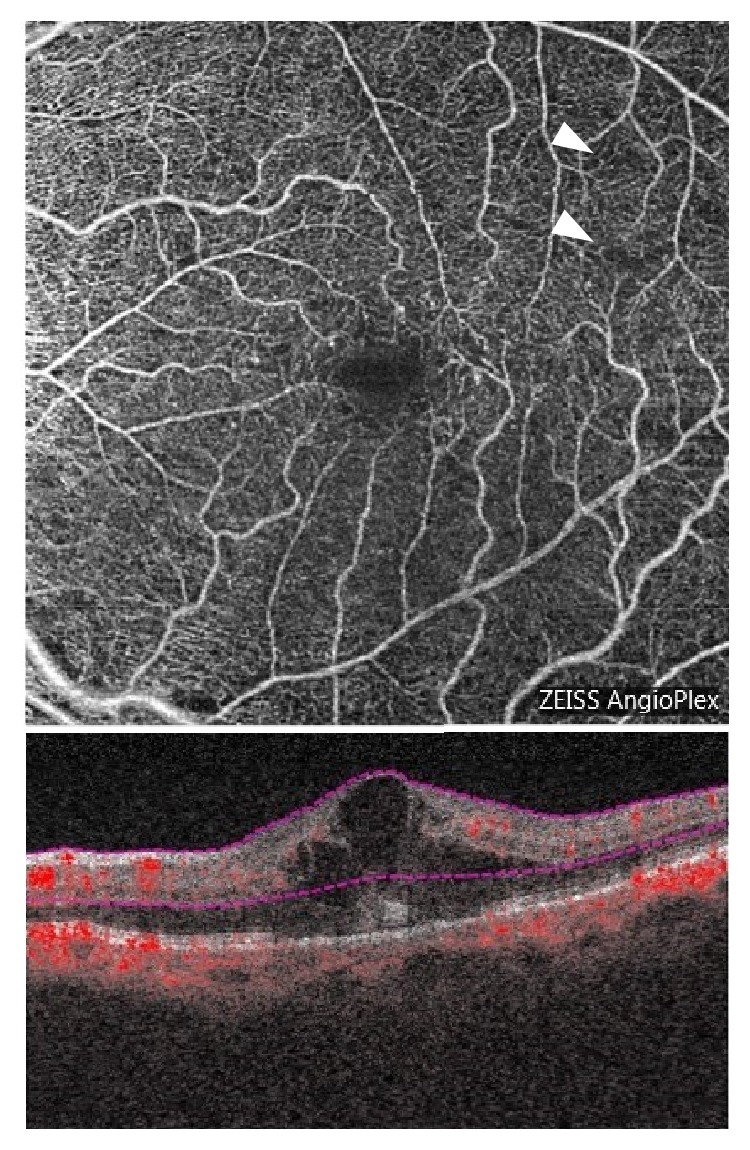
An OCTA image of the superficial capillary plexus in the left eye shows loss of small capillaries (arrowheads) in the upper right region.

An 81-year-old woman was referred to our Retina Clinic because of loss of visual acuity (VA) in the left eye 3 months ago. She had a history of uncontrolled hypertension, hyperlipidemia, and diabetes mellitus. Her best-corrected visual acuity (BCVA) was 20/20 in the right eye and 20/30 in the left eye. Funduscopic examination of the left eye showed typical features of nonischemic CRVO, including venous tortuosity and intraretinal hemorrhage in all four quadrants of the fundus. Fluorescein angiography (FA) and OCTA showed loss of small capillaries but nonperfusion areas were not observed, confirming a diagnosis of nonischemic CRVO. Optical coherence tomography (OCT) showed retinal thickening in the fovea with associated inner retinal cysts. (Figures 1 and 2)

**Figure 3:**
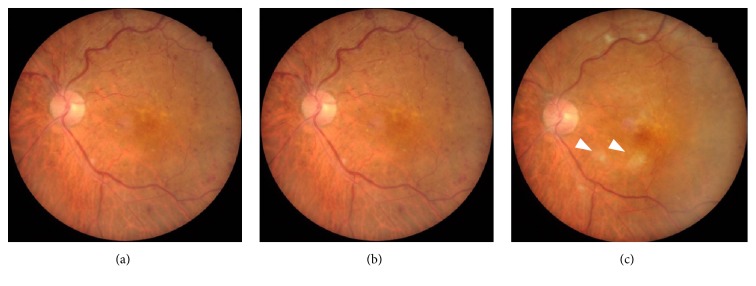
Fundus photograph images in the left eye (a) 1 month, (b) 2 months, and (c) 4 months after the initial visit. (c) Fundus photograph demonstrating cotton wool spots (arrowheads) located near the macula.

**Figure 4:**
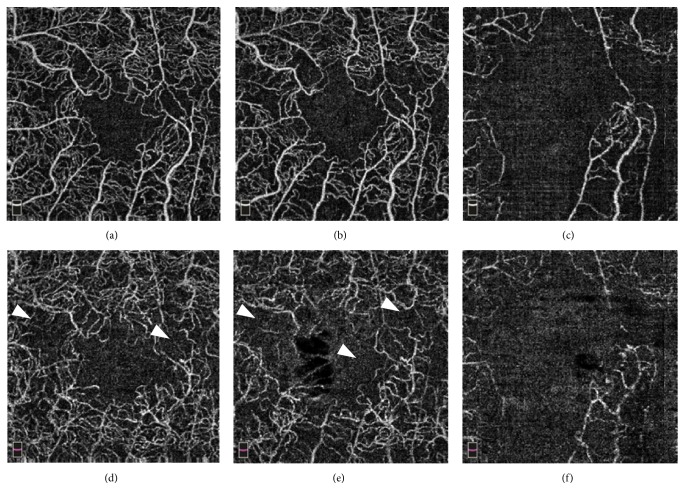
OCTA images of the SCP in the left eye (a) 1 month, (b) 2 months, and (c) 4 months after the initial visit. OCTA images of the DCP in the left eye (d) 1 month, (e) 2 months, and (f) 4 months after the initial visit. OCTA images demonstrating expanding nonperfusion area in both SCP and DCP. In DCP, extensive avascular areas are recognized earlier than in SCP (arrowheads).

**Figure 5:**
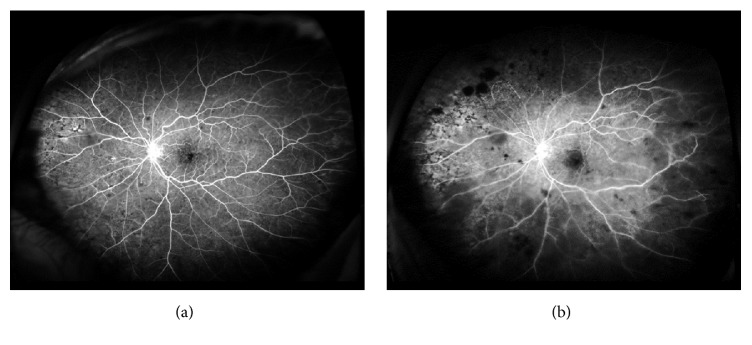
Fluorescein angiography image in the left eye at (a) initial visit demonstrating loss of small capillaries, but nonperfusion areas werenot observed. (b) 6 months after the initial visit demonstrating more than 10 discs areas of nonperfusion.

**Figure 6:**
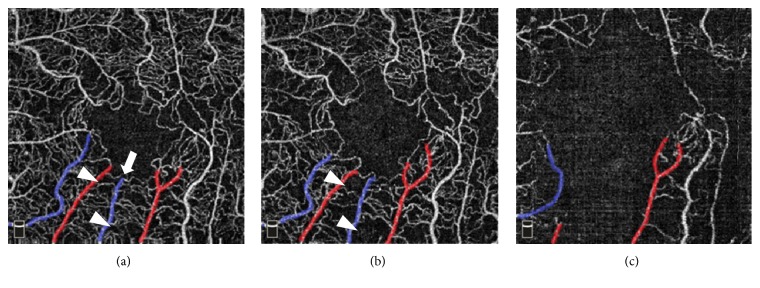
OCTA images in the left eye (a) 1 month, (b) 2 months, and (c) 4 months after the initial visit. The artery is colored red and the vein is colored blue. Detailed sequential OCTA observations reveal progressive capillary dropout from the areas around the vein (arrow), followed by disappearance of the vein surrounded by avascular areas (arrowheads).

Intravitreal injection of the antivascular endothelial growth factor (VEGF) drug aflibercept was initiated. One month later, visual acuity was improved to 20/25 along with gradual normalization of fundus findings associated with CRVO and macular cysts. After one more month OCTA revealed enlargement of the foveal avascular zone and broken foveal capillary ring in the superficial capillary plexus (SCP) and deep capillary plexus (DCP). Color fundus photograph demonstrated cotton wool spots located near the macula (Figure 3); OCTA revealed gradual enlargement of the avascular zone in SCP and DCP; and visual acuity was impaired. Extensive avascular areas were observed in the DCP earlier than in the SCP (Figure 4). Nine months after onset of symptoms, FA showed more than 10-disc area of retinal capillary nonperfusion (Figure 5). Conversion from nonischemic CRVO to ischemic CRVO was diagnosed. In addition, detailed sequential observations with OCTA revealed progressive capillary dropout from areas around the vein, forming avascular areas. At a specific timeframe during follow-up, the vein surrounded by avascular areas showed loss of patency (Figure 6). There was no neovascularization.

## 3. Discussion

We performed sequential OCTA observations on a case initially presented with nonischemic CRVO which subsequently converted to ischemic CRVO. OCTA demonstrated capillary dropout occurring preferentially around the vein and these areas gradually enlarged forming avascular lesions. Moreover, OCTA revealed more extensive avascular zones in the DCP than in the SCP. Previous study has also reported that retinal vascular abnormalities in diseases of retinal veins develop more frequently in the DCP than in the SCP [5]. Gradually enlargement of retinal capillary dropout eventually led to the conversion from nonischemic CRVO to ischemic CRVO. The conversion to ischemic CRVO occurred stepwise rather than abruptly.

In conclusion, the present case suggests that conversion to ischemic CRVO starts in the areas around the vein and progresses stepwise rather than abruptly. Moreover, in OCTA study, avascular zones are more readily recognized in the DCP than in the SCP. This is the first report of sequential observations of conversion from nonischemic CRVO to ischemic CRVO using OCTA. Further study of more cases may contribute to elucidation of the mechanism of conversion to ischemic CRVO.
